# Learning Protein–Protein Binding Free Energies from Interface Graphs and Physicochemical Descriptors

**DOI:** 10.1021/acsphyschemau.6c00060

**Published:** 2026-08-01

**Authors:** Qingshu Zhao, Arjun Saha

**Affiliations:** 118733 The University of Wisconsin-Milwaukee, Milwaukee, Wisconsin 53211, United States

**Keywords:** protein−protein interactions, machine learning, graph neural networks, binding free energy, high-throughput screening

## Abstract

Predicting protein–protein binding free energy (ΔG) from structure remains a central challenge in computational biophysics. Here, we present GULP (Graph-based Unified Learning for Protein binding), a graph neural network (GNN) that jointly learns from a residue-level graph representation of the binding interface and global physicochemical descriptors. We systematically investigate how training data distribution affects model performance by comparing a full training set with a balanced subset enriched for extreme-affinity complexes. GULP is computationally efficient and provides interpretable insights into residue-level and physicochemical contributions to binding. On external validation, GULP achieves a mean absolute error (MAE) of 2.31 kcal/mol and shows moderate agreement with experimental ΔG values (Pearson r = 0.54, Spearman ρ = 0.58).

## Graphical abstract

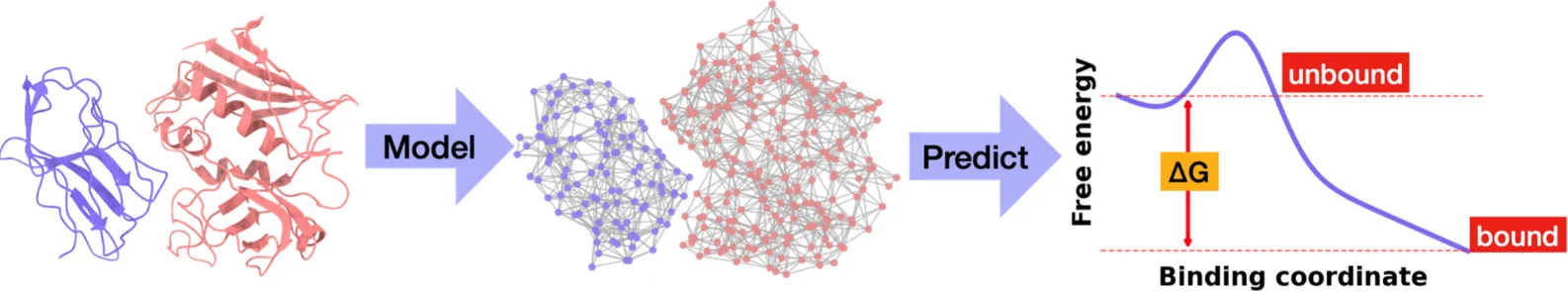

Protein–protein interactions (PPIs) are fundamental to virtually all cellular processes, from signal transduction and immune recognition to metabolic regulation and disease pathogenesis.^1–4^ Accurately predicting PPI binding affinity has broad applications in therapeutic antibody design, drug discovery, and protein engineering.^1,3,5–7^ Although experimental techniques such as isothermal titration calorimetry and surface plasmon resonance can measure binding affinities with high precision, they are costly and low throughput, which motivates the development of computational approaches.

Physics-based methods such as free energy perturbation (FEP)^8^ and molecular mechanics generalized Born surface area (MM-GBSA)^9^ rely on molecular dynamic simulations^10^ with prohibitive computational costs. Statistical and machine learning (ML) approaches have emerged as faster alternatives, but existing models tend to fall into one of two categories, each with significant limitations. The first comprises models that rely heavily on physics-based energy function descriptors, such as Rosetta-based scoring terms.^11–14^ While physically grounded, such approaches require complex preprocessing pipelines and limit practical accessibility for users. The second comprises data-driven deep learning models trained on large data sets,^15–17^ which can capture complex structural patterns but suffer from low interpretability and are prone to overfitting noise, making it difficult to extract mechanistic insight from their predictions.

Improving learning on PPI complexes requires not only appropriate model design but also careful consideration of training data distribution and evaluation protocols. Existing protein–protein binding affinity data sets exhibit strong distributional bias, with most complexes clustered near moderate binding strengths.^18–21^ This imbalance can drive models toward predicting near-average values and limits their ability to accurately capture extreme binders, which are often of greatest practical interest. In addition, structural redundancy within available data sets can lead to overly optimistic performance estimates when similar complexes appear in both training and evaluation sets.

Together, these limitations highlight the need for a prediction framework that is (i) computationally efficient, (ii) physically interpretable, and (iii) robust to data set bias and structural redundancy. To address these challenges, we present GULP (Graph-based Unified Learning for Protein binding), a graph neural network (GNN) framework that learns jointly from residue-level interface graphs and physicochemical descriptors to predict protein–protein binding free energy.

The framework combines two complementary sources of information. A residue-level graph captures the structural context of the protein–protein interface, while physicochemical descriptors provide physically interpretable features associated with binding. For each protein–protein complex, physicochemical descriptors are first extracted. These descriptors include interface properties such as number of interfacial hydrogen bonds, atomic contacts, salt bridges, buried surface area, and various noninterface surface (NIS) properties (Table S1). The complex is also represented as a residue-level graph (Figure S1), where each residue is embedded with its amino acid identity and class of polarity, and the extracted physicochemical descriptors are incorporated as global graph features. Residues at the binding interface, nearby noninterface surface (NIS shell), and distal noninterface surface (NIS distal) are encoded in a zone-defined graph, allowing GULP to distinguish direct binding interactions from the surrounding structural context. The residue graph is processed by two graph convolution layers to learn a structural representation of the protein–protein interface, and the combined information is processed by a small multilayer perceptron to predict the binding free energy (ΔG) (Figure 1). Full details of data preprocessing, feature construction, and model training are provided in the Supporting Information.

**Figure 1:**
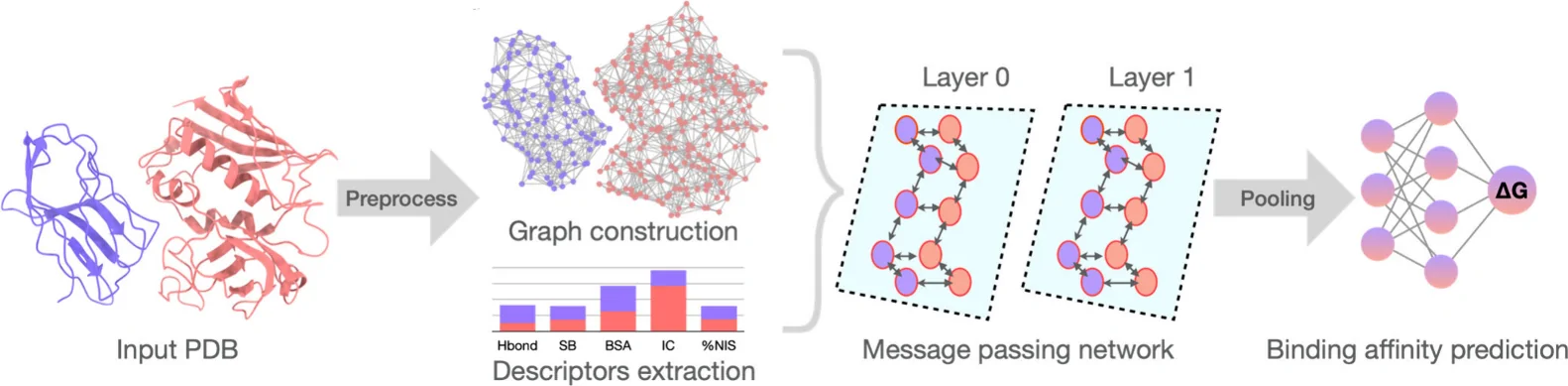
GULP binding affinity prediction workflow. The input PDB structure is converted into a residue-level graph, with physicochemical descriptors incorporated as global graph features. This graph is then processed by a GNN consisting of two graph convolution layers, followed by a small multilayer perceptron that predicts the binding free energy ΔG.

To train and evaluate the proposed framework, we curated a high-quality data set of experimentally characterized PPI complexes from the PPB-Affinity database^22^ (Figure S2). Only wild-type complexes were retained, and duplicate entries were removed before applying additional structure-quality filters. Complexes with more than 20% missing residues or more than 70% coil content were excluded to minimize the inclusion of intrinsically disordered proteins. Structures containing nonstandard ligands/cofactors (such as ATP, ADP, GDP, GTP, FMN, heme-related groups, or biotin) were removed, as they can alter protein conformations and binding affinity. Dissociation constants were converted to ΔG using ΔG = −RT ln *K*D at the recorded experimental temperature (or 298 K when unavailable).

The resulting training set (n = 517) exhibits a strong bias toward moderate affinities, with most complexes clustered near – 10 kcal/mol (Figure 2). To examine the effect of data distribution, we constructed a second, more balanced training set with strong and weak binders (n = 202). An external test set of 98 complexes, drawn from the same structurally filtered pool, was held out for evaluation.

**Figure 2:**
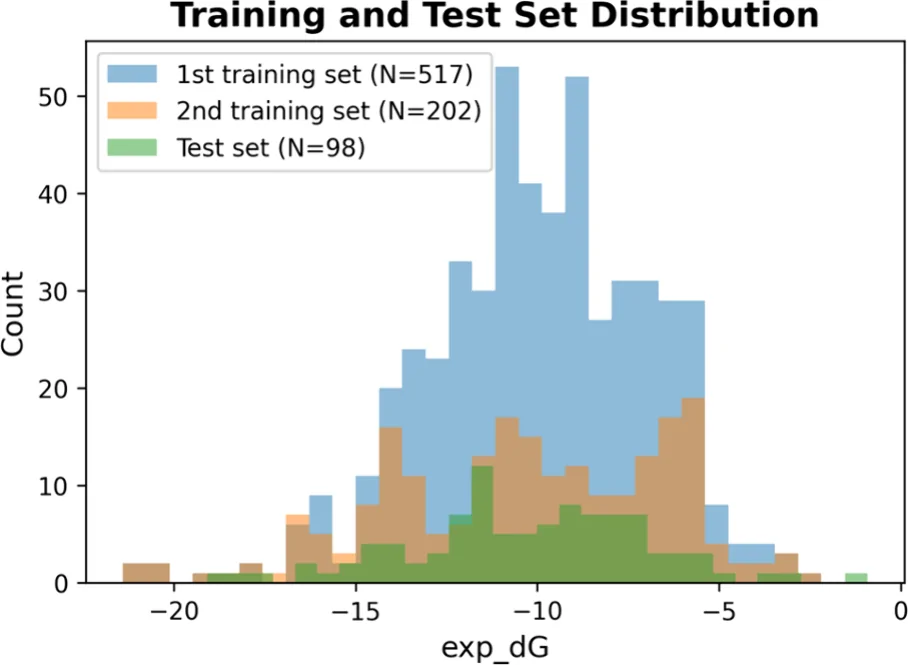
Experimental binding free energy distributions of the training and test set. The first training set of structurally filtered dimeric PPI complexes follows Gaussian distribution peaked around −10 kcal/mol. The second training set was constructed to achieve a markedly even coverage across the full affinity range.

Model performance was first evaluated by cross-validation on the full training set using random train-test split and further examined across different levels of sequence similarity between the training and the test sets according to their Smith-Waterman similarity score.^23^ As expected, random train-test split produced the highest performance (Figure 3), whereas GULP progressively declined as the sequence similarity threshold became more stringent. Nevertheless, GULP consistently outperformed individual physicochemical descriptors when tested on dissimilar sequences (Pearson r = 0.38 vs 0.26), suggesting that it learns transferable structural patterns rather than simply memorizing structures.

**Figure 3:**
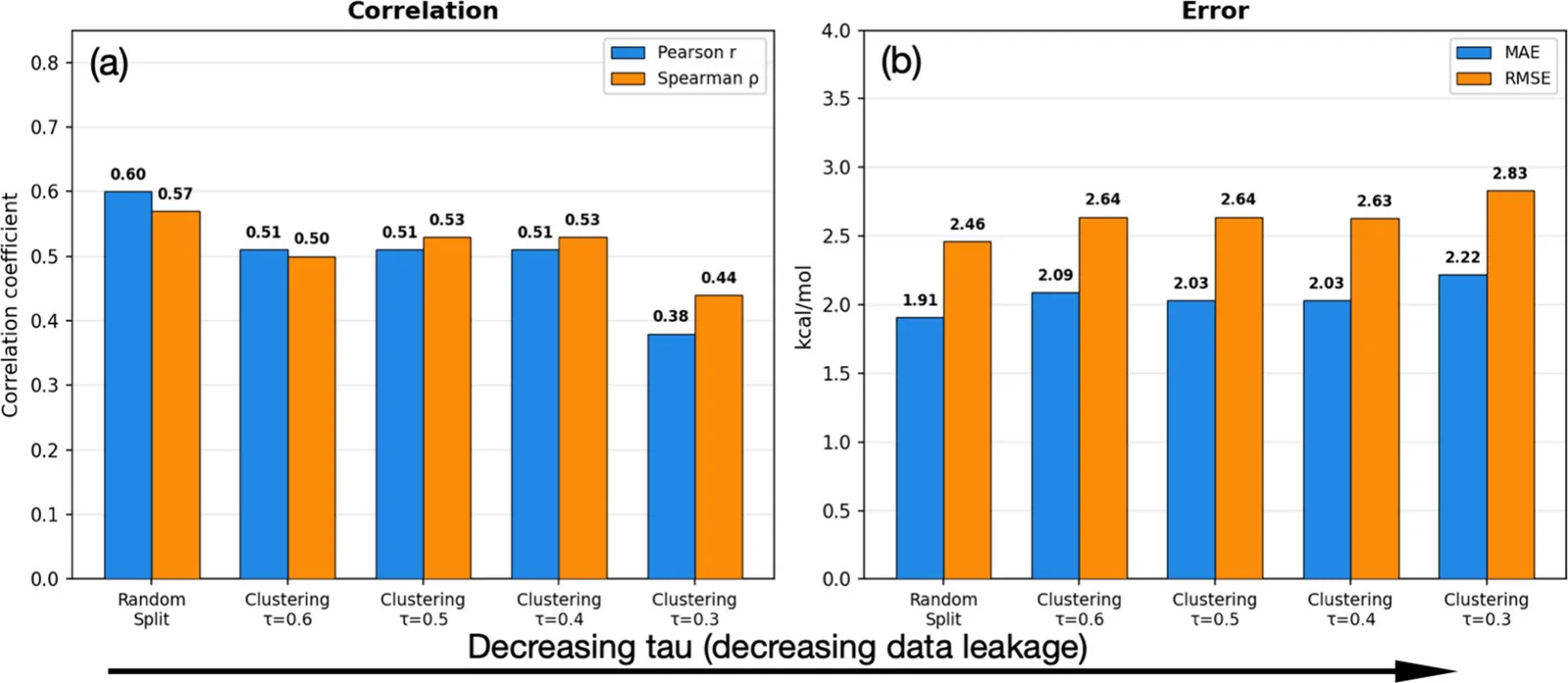
Internal model performance under varying data leakage. Correlation (a) and error (b) are shown for random split and clustering-based cross-validation at four sequence identity thresholds (τ = 0.3–0.6). Performance degrades as the clustering threshold becomes more stringent.

Having established that conventional evaluation can overestimate performance, we next assessed model behavior on an external test set. We compared our model against PRODIGY v2.4.0,^13^ a widely used physics-based method for PPI binding affinity prediction. PRODIGY predictions were generated using default settings on the same 98-complex external test set. Our proposed model shows improved performance in high-similarity regimes (r = 0.54, RMSE = 3.04) (Figure 4a), outperforming PRODIGY (r = 0.15, RMSE = 3.68). As sequence similarity to the training set decreases, model performance declines, with correlation dropping to r = 0.38 in the medium-similarity regime and r = 0.37 in the low-similarity regime. Despite this degradation, GULP maintains moderate predictive correlation and consistently outperforms PRODIGY across all similarity bins. More broadly, the performance observed here falls within the range reported for recent ML-based PPI affinity predictors, which typically achieve Pearson correlations of approximately 0.4–0.7 ^15,24–26^ depending on the test data set and evaluation strategy [Table S3]. Although direct comparisons are difficult because different studies employ different data sets and model architectures, the correlations obtained in our work should be interpreted in the context of a challenging and realistic setting.

**Figure 4:**
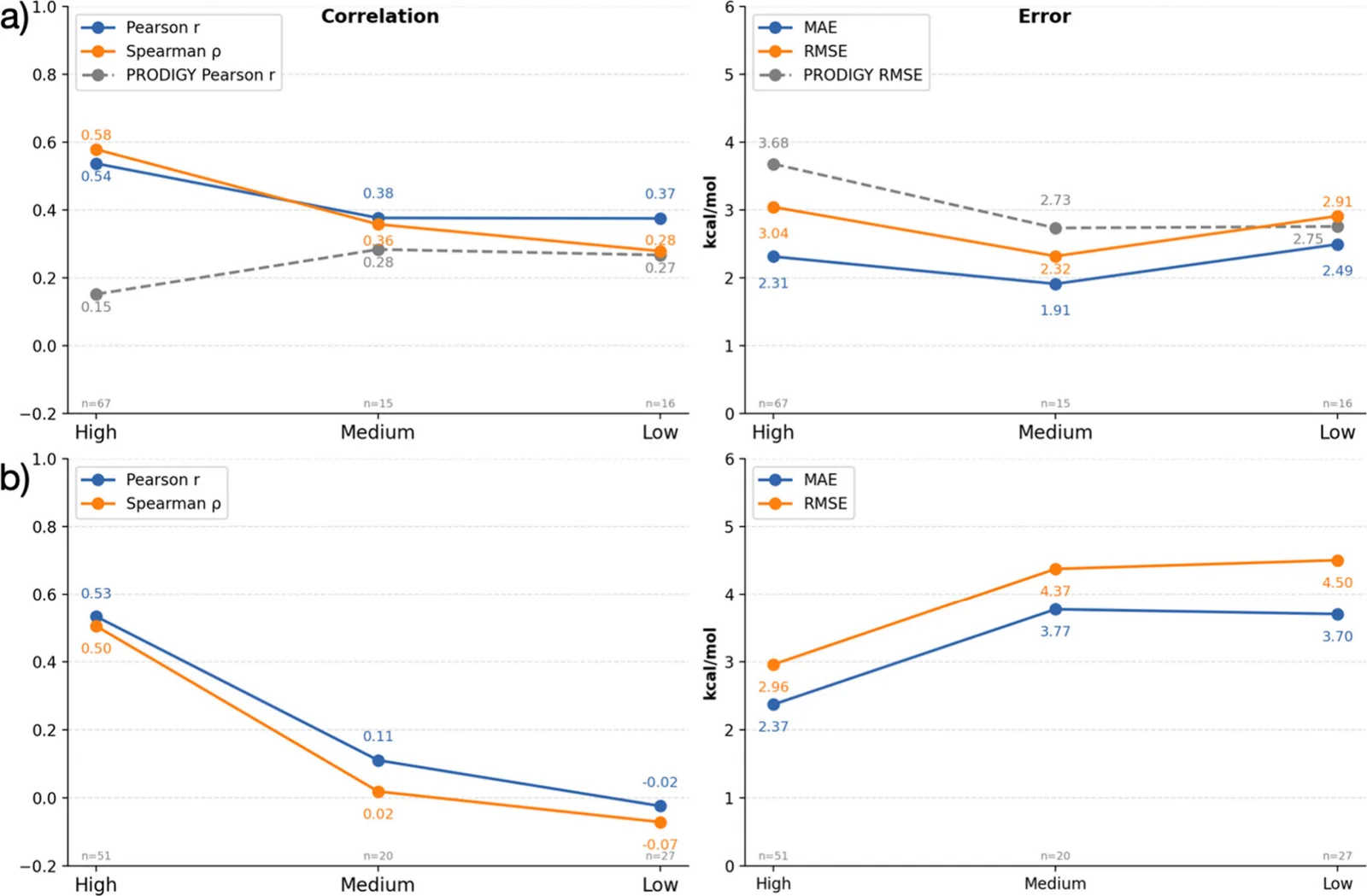
External validation performance grouped by sequence similarity to the training set. Prediction accuracy for binding free energy is shown across high, medium, and low sequence similarity bins relative to the nearest training complex. (a) Model trained on the full data set; (b) model trained on the distribution-balanced data set. Left panels report correlation (Pearson r and Spearman ρ), and right panels report error (MAE and RMSE). Benchmark comparison with PRODIGY is shown in dotted line in (a).

To assess the contribution of the physicochemical descriptors, we performed an ablation analysis by training the GNN with different subsets of physicochemical features and evaluating each model on the external test set (Table S4, Figure S4). Using the GNN embeddings alone yielded the weakest performance (r = 0.37). Adding a subset of physicochemical descriptors improved performance (r = 0.42–0.46), but the best results were consistently achieved when all seven descriptors were included. This suggests that each descriptor contributes useful information, complementing the structural features learned by the GNN.

We next evaluated whether modifying the training data distribution could further improve generalization on the external test set. Compared to the model trained on the full data set, model trained on a balanced data set shows similar performance for structurally similar complexes (Figure 4 and Figure S5). Both models exhibit decreasing accuracy as sequence similarity to the training set decreases. However, the model trained on a balanced data set degrades more substantially for mid- and low-homology complexes, particularly in the midrange binding regime (ΔG ≈ −7 to −11 kcal/mol). This likely reflects that the balanced data set contains fewer midrange complexes, providing less training reference for predictions. In contrast, the balanced model improves predictions for several strong binders (e.g., 1BRS, 2PSM, 6KBR), with errors reduced by >2 kcal/mol (Figure S5), consistent with the intentional oversampling of extreme-affinity complexes. These results highlight a trade-off between distributional balance and data set size: balanced sampling improves extreme predictions but reduces stability in densely populated affinity regions.

To better understand how GULP makes predictions across these distributions, we performed an attribution analysis of the GNN using Integrated Gradients^27,28^ (Figure 5). Residues at the binding interface show the largest contribution to the prediction, followed by nearby surface residues (NIS shell), while distant residues (NIS distal) contribute less (Figure 5a). At the node-feature level, GULP relies on amino acid identity and residue type, with polar and apolar residues and specific amino acids such as serine, leucine, and alanine contributing most strongly (Figure 5b). This pattern is consistent with established observations in protein–protein interactions, where residues such as serine and leucine frequently occur at binding interfaces,^29–31^ and polar–hydrophobic patterning plays an important role in governing binding specificity.^32–34^ In addition, features related to interface contacts, surface composition, and buried surface area are among the most important predictors (Figure 5c). These results indicate that GULP captures chemically sound signals and provides a basis for understanding where the model performs well and where it fails.

**Figure 5:**
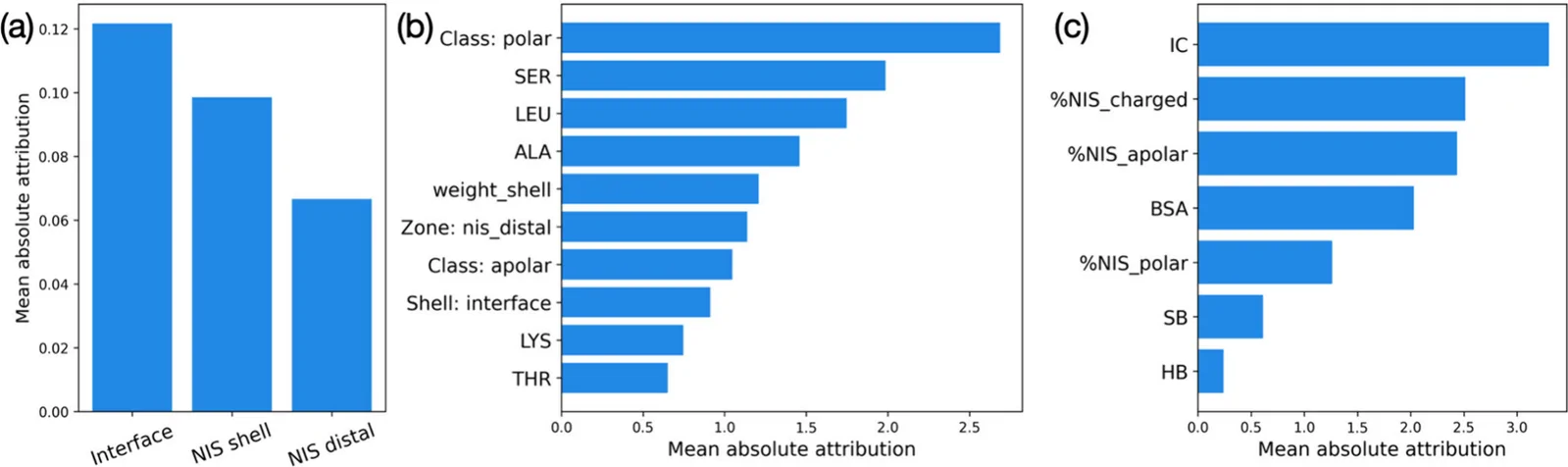
Attribution analysis of GULP’s predictions on test set. (a) Mean absolute attribution per residue averaged by structural zone. Interface residues contribute most strongly, followed by nearby noninterface surface residues (NIS shell) and distal noninterface residues (NIS distal) (b) Top residue-level features ranked by mean absolute attribution. Residue class (e.g., polar), residue identity (e.g., serine, leucine), and spatial context (zone and shell weighting) are key contributors to model predictions. (c) Physicochemical features ranked by mean absolute attribution. Interfacial contacts (IC) and NIS composition (% charged residues and % apolar residues) are dominant determinants, with buried surface area (BSA) and number of salt bridges (SB) contributing moderately and number of hydrogen bonds (HB) playing a smaller role.

Despite these insights, GULP exhibits systematic errors for certain classes of complexes. To understand these failures, we analyzed representative outliers in the external test set (Figure S5). Prediction outliers clustered into several interaction classes rather than arising randomly. Highly optimized inhibitor–enzyme complexes, including trypsin/BPTI-like systems and BLIP-I/TEM-1 β-lactamase (2PTC, 3FP6, 3GMW, and 1MCV),^35–38^ likely challenge the model because their affinities depend on precise atomic packing, shape complementarity, and low entropic penalties that are only partially represented by residue-level graphs. 6IVU,^39^ a solution NMR structure, highlights a second limitation: static graph representations cannot capture ensemble-averaged interactions or conformational heterogeneity. A third class includes prolactin receptor complexes (3NCC, 3MZG, and 3N06),^40^ whose binding has been reported to depend strongly on interface histidine and solution acidity, suggesting missing protonation-state effects. Finally, 4A49 ^41^ represents a regulated ubiquitination complex in which binding is coupled to phosphorylation-induced conformational activation, indicating that allosteric state dependence may also contribute to model error. These cases suggest that the largest errors occur for systems involving extreme affinities, unusual interface classes, or binding energetics strongly influenced by dynamics, protonation, or regulation.

While these results provide insight into GULP’s decision-making process, they also highlight several limitations in the underlying data and modeling framework. Because the number of experimentally measured complexes is limited, increasing the training set often reduces the structural novelty of validation sets, which complicates rigorous generalization assessment. This was experienced in our study, where the limited and sparsely distributed number of strong (<−16 kcal/mol) and weak (>−5 kcal/mol) binders restricts GULP’s ability to distinguish between these regimes. In addition to data limitations, noise remains difficult to control. Variability can arise from experimental measurements or from intrinsically atypical protein–protein interactions. While incorporating diverse complexes is desirable, combining different interaction types (e.g., enzyme–inhibitor and antibody–antigen) may obscure interaction-specific patterns. Increasing model complexity may further amplify this issue; for example, atom-level graph representations could capture finer structural detail but also increase the risk of overfitting to noisy features. Finally, our model is restricted to dimeric complexes, which simplifies the representation but limits applicability to multimeric systems where cooperative effects are important.

We demonstrate that competitive performance can be achieved with an interpretable model, with evaluation conducted under realistic conditions. Importantly, our findings suggest that current limitations in binding affinity prediction are driven less by model capacity and more by data set composition, particularly the uneven distribution of binding affinities. Future improvements will likely depend on distribution-aware data curation and more informative representations rather than increased model complexity alone.

## Supplementary Material



## Data Availability

All curated data sets and source code required to reproduce the analyses presented in this work are publicly available at: https://github.com/SahaLabGitHub/GULP.

## Notes

Additional figures and tables, model details, and references (PDF)
