# Supplementary material for: Learning Protein–Protein Binding Free Energies from Interface Graphs and Physicochemical Descriptors
Source: ACS Phys Chem Au. 2026 Aug 1;6(5):1012–7. doi: 10.1021/acsphyschemau.6c00060 (PMC13614133; doi:10.1021/acsphyschemau.6c00060)
Supplement: Supplementary file 1 [file pg6c00060_si_001.pdf]

Learning Protein–Protein Binding Free Energies from Interface Graphs and Physicochemical Descriptors

Support Information

Qingshu Zhao[†], Arjun Saha[†]*

*Corresponding author email: saha6@uwm.edu

[†]Author affiliation: Department of Chemistry and Biochemistry, University of Wisconsin-Milwaukee, Milwaukee, Wisconsin 53211, United States

Table of Contents

Section 1: Figures (page 2-5)

Section 2: Tables (page 6-11)

Section 3: Model details (page 12-13)

Section 4: References (page 14)

Section 1: Figures

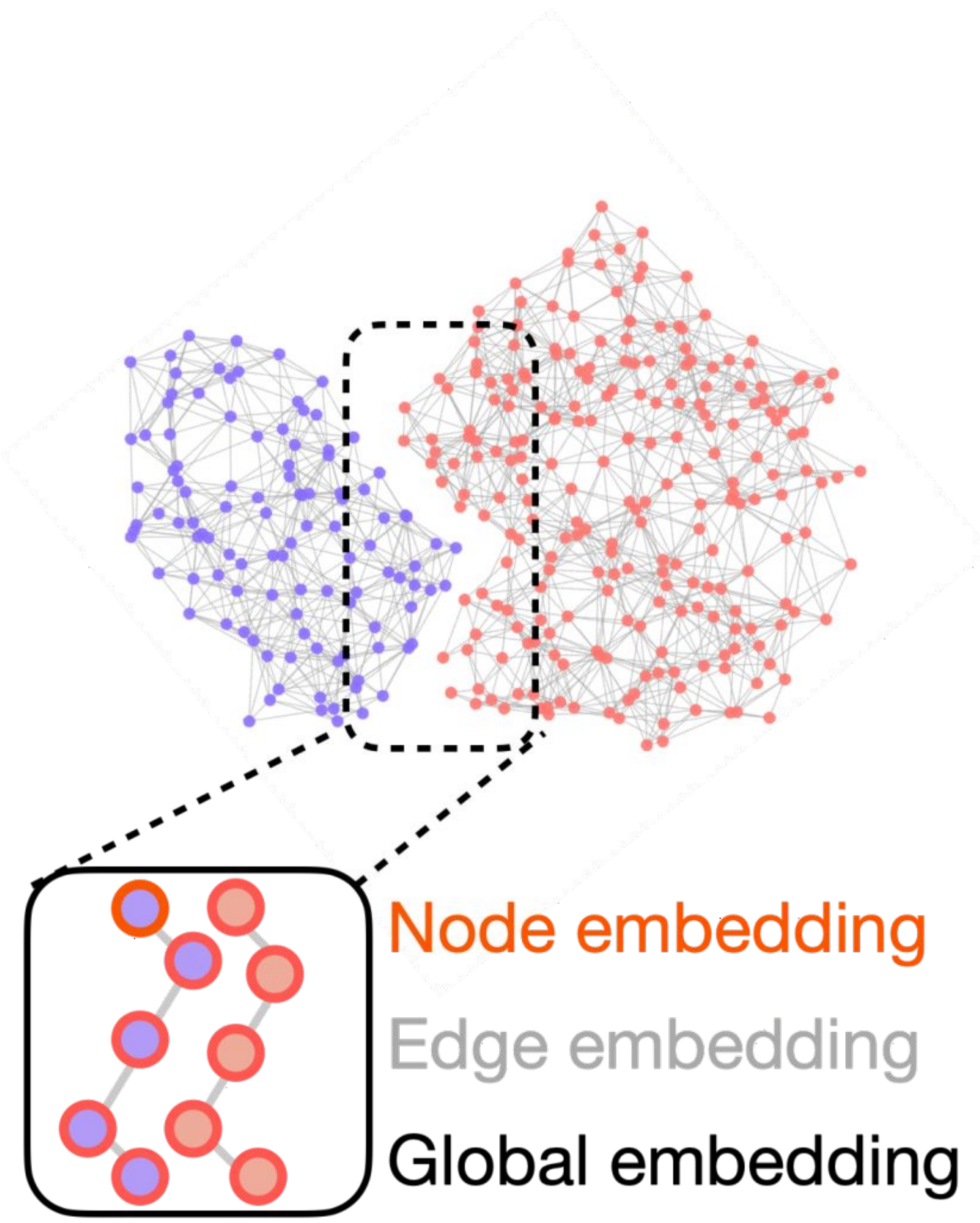

Figure S1. Residue-level graph of the protein-protein complex

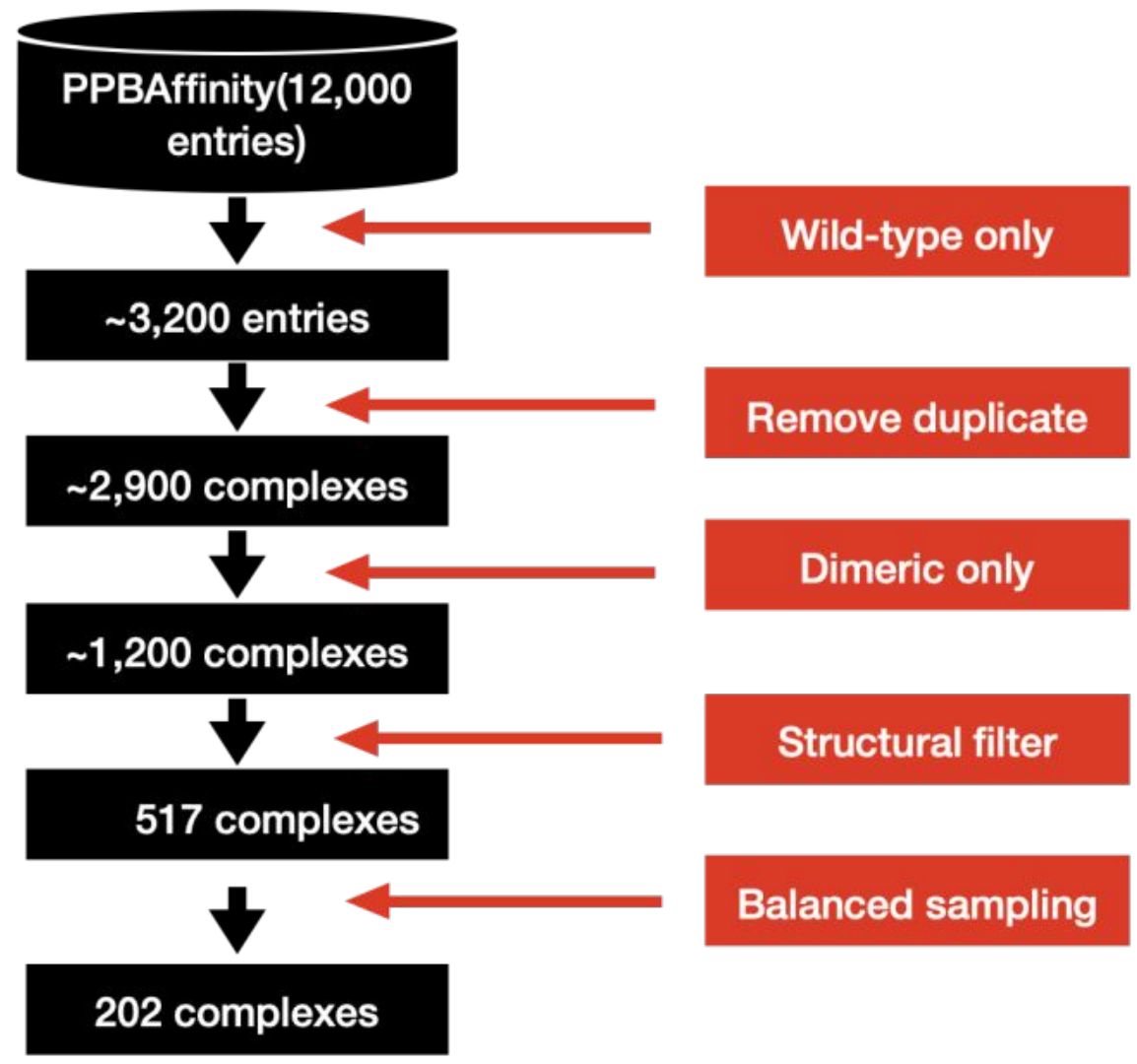

Figure S2. Training dataset curation pipeline. Starting from the PPB-Affinity database, wild-type complexes were retained, and duplicate entries removed. These unique entries were then filtered to dimeric complexes only, followed by a structural quality filter to produce a pool of 517 candidates. From this pool, we constructed a second, more balanced training subset with improved coverage of strong and weak binders.

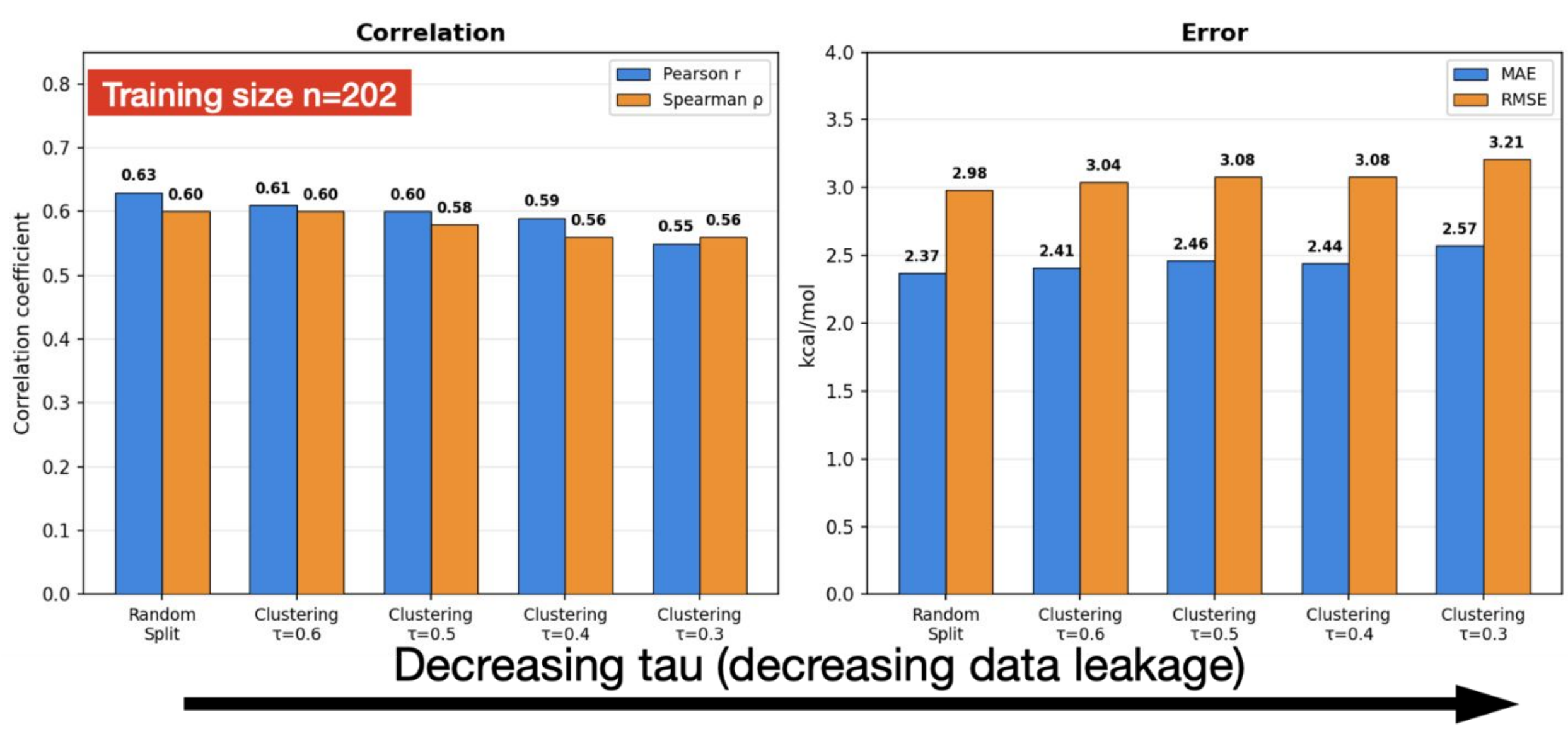

Figure S3. Internal model performance under varying data leakage. Correlation (a) and error (b) are shown for random split and clustering-based cross-validation at four sequence identity thresholds (τ = 0.3–0.6). Model performance degrades as the clustering threshold becomes more stringent.

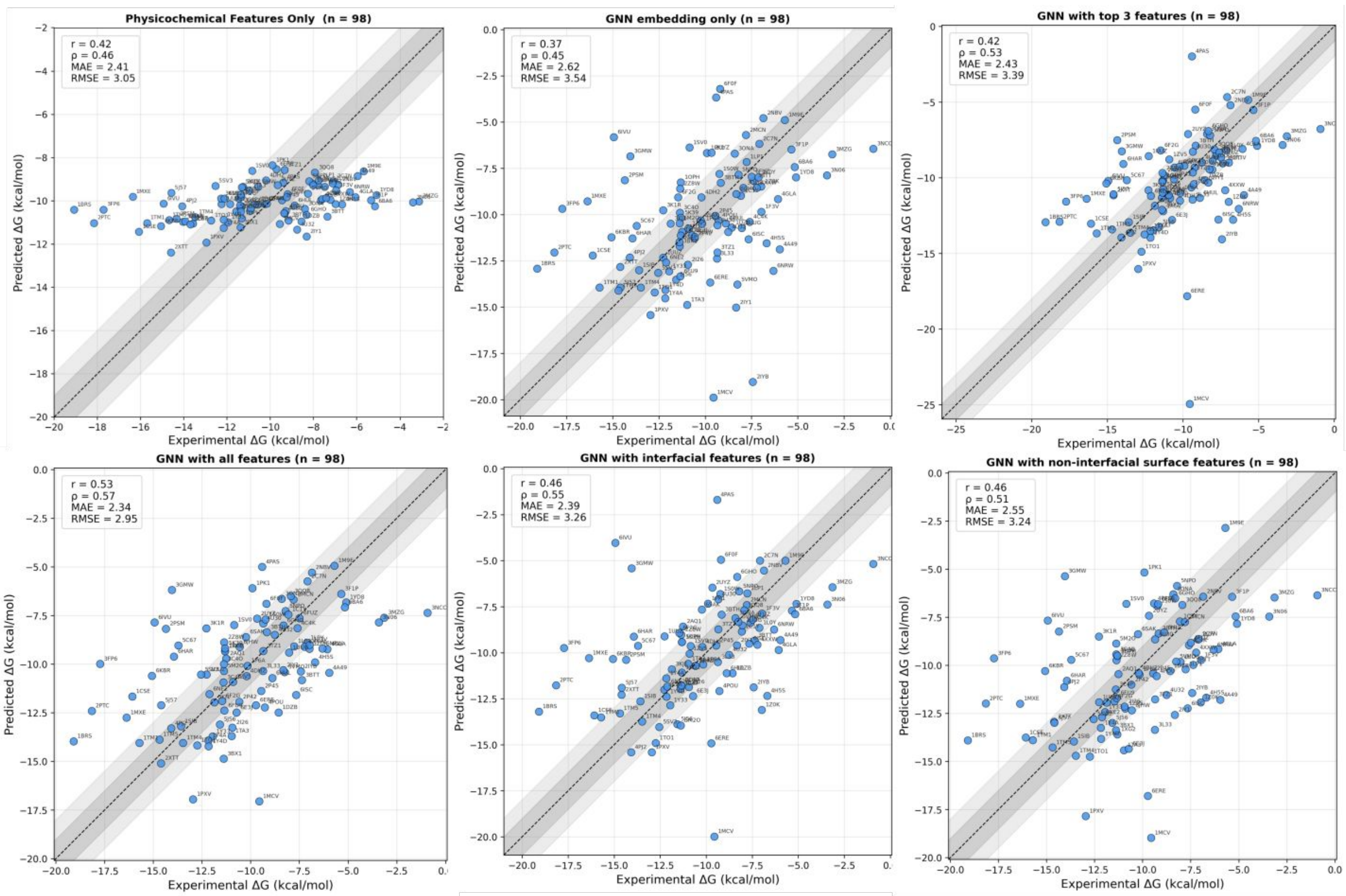

Figure S4. External test performance of models trained with different feature sets. Scatter plots of predicted vs. experimental binding free energies ΔG for models using physicochemical descriptors only, GNN embeddings only, and various combinations of physicochemical and graph embeddings.

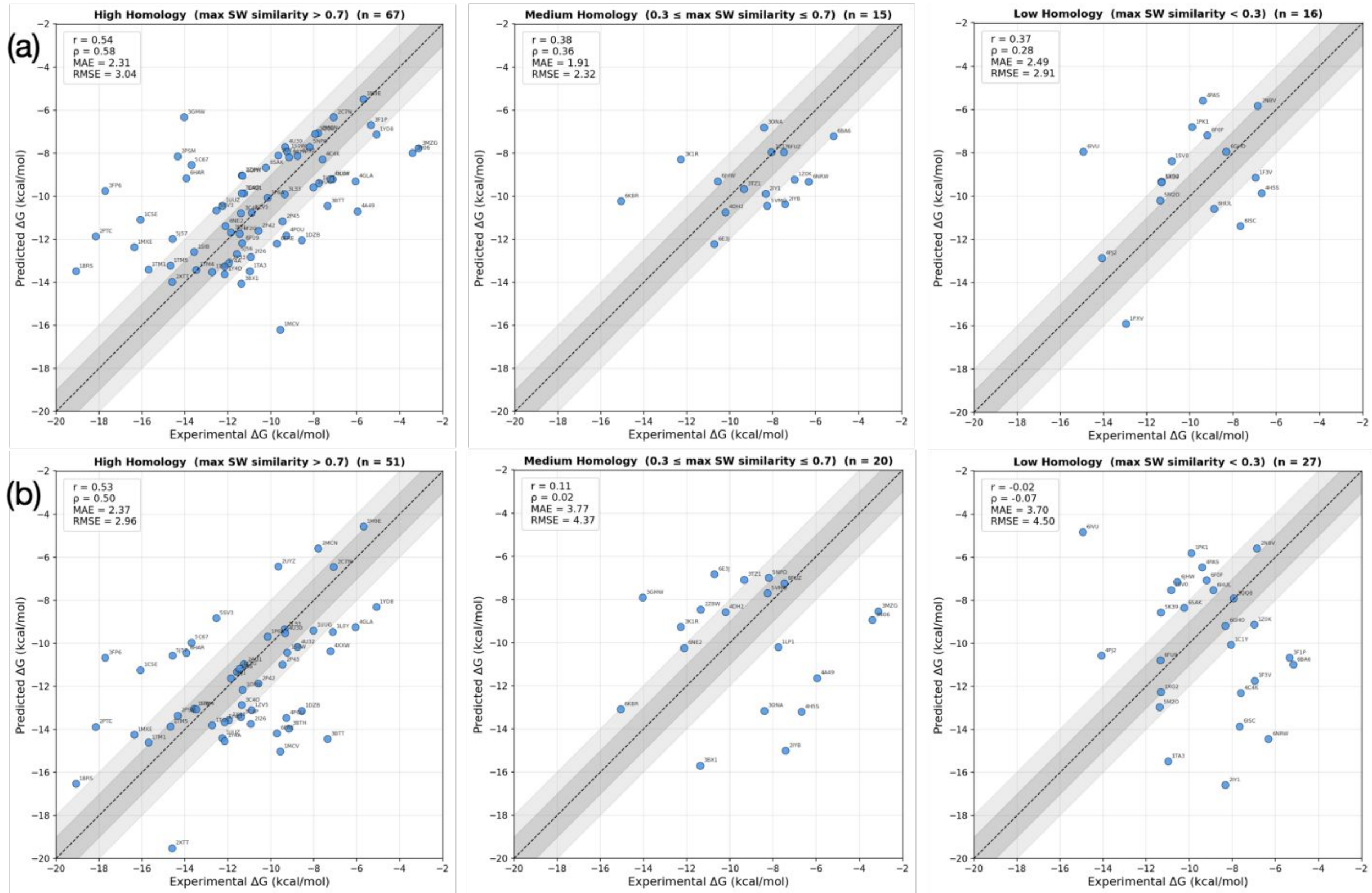

Figure S5. External validation performance grouped by sequence similarity to the training set. Scatter plots of predicted vs. experimental ΔG (kcal/mol) for the model trained on **(a)** full training set and model trained on the **(b)** balanced training set, separated by sequence similarity to the nearest training structure.

Section 2: Tables

Table S1. Pearson's correlations between experimental binding affinities and the physio chemical features in training dataset.

Features	Pearson
Interfacial contacts (IC)	–0.26
number of interfacial hydrogen bonds (HB)	–0.22
percentage of polar residues at non-interface surface (%NIS_polar)	–0.19
Buried surface area (BSA)	–0.17
percentage of charged residues at non-interface surface (%NIS_charged)	+0.13
number of interfacial salt bridge (SB)	–0.09
percentage of apolar residues at non-interface surface (%NIS_apolar)	–0.07

Features were computed directly from input PDB structures using a combination of Bio.PDB[1], MDAnalysis[2,3], and FreeSASA[4]. All features were calculated between the two protein chains identified in each complex. Following is the definition of each feature:

Interfacial contacts (IC): Interfacial contacts were defined as the number of residue–residue pairs across chains that exhibit at least one heavy-atom contact within 5.5 Å. Heavy atoms from both chains were extracted, and all inter-chain residue pairs within this distance cutoff were identified using a neighbor search algorithm. The total number of such contacts was reported as IC.

Number of interfacial hydrogen bonds (HB): Hydrogen bonds across the protein–protein interface were computed using MDAnalysis HydrogenBondAnalysis. When hydrogen atoms were present in the structure, hydrogen bonds were identified using geometric criteria: donor–acceptor distance $\leq$ 3.89 Å, hydrogen–acceptor distance $\leq$ 4.0 Å, and donor–hydrogen–acceptor angle $\geq$ 90°. Only hydrogen bonds formed between residues from different chains were counted. For structures lacking explicit hydrogen atoms, a heavy-atom distance-based approximation was used, counting donor–acceptor pairs within the same distance cutoff.

Buried surface area (BSA): Buried surface area was calculated using FreeSASA as:

$$BSA = (SASA_A + SASA_B - SASA_{complex})/2$$

where $SASA_A$ and $SASA_B$ are the solvent-accessible surface areas of the individual chains, and $SASA_{complex}$ is that of the bound complex. Each chain was extracted and evaluated independently to compute its contribution.

Number of interfacial salt bridge (SB): Salt bridges were defined as electrostatic interactions between oppositely charged residues across chains, specifically between:

- Positively charged atoms: Lys (NZ), Arg (NE, NH1, NH2)
- Negatively charged atoms: Asp (OD1, OD2), Glu (OE1, OE2)

All cross-chain atom pairs within 4.0 Å were identified, and each qualifying interaction was counted as a salt bridge.

Non-Interface Surface Composition (%NIS_polar, %NIS_charged, %NIS_apolar): Residues were first classified based on relative solvent accessibility (RSA) computed using FreeSASA. Residues with RSA ≥ 0.05 were considered surface residues. Among these, residues not participating in interfacial contacts were defined as non-interface surface (NIS) residues. NIS residues were categorized into three classes:

- Polar: SER, THR, ASN, GLN, TYR, CYS, TRP, HIS
- Charged: ASP, GLU, LYS, ARG
- Apolar: ALA, VAL, ILE, LEU, MET, PHE, PRO, GLY

The percentage of each class was computed relative to the total number of NIS residues:

$$\%NIS_{class} = (N_{class}/N_{total})*100$$

where N_{class} is the number of residues in each category.

Table S2. PRODIGY's predication on test set

PDB	exp_dG	PRODIGY_pred_dG
**1BRS**	-19.09	-11.32
**2PTC**	-18.16	-12.27
**3FP6**	-17.73	-10.97
**1MXE**	-16.37	-10.54
**1CSE**	-16.08	-11.37
**1TM1**	-15.71	-11.72
**6KBR**	-15.07	-12.39
**6IVU**	-14.94	-10.79
**1TM5**	-14.69	-11.66
**2XTT**	-14.60	-13.09
**5J57**	-14.59	-8.56
**2PSM**	-14.35	-10.59
**4PJ2**	-14.08	-11.06
**3GMW**	-14.05	-12.77
**6HAR**	-13.94	-11.23
**5C67**	-13.71	-11.20
**1SIB**	-13.58	-12.19
**1TM4**	-13.48	-11.63
**1PXV**	-12.96	-13.96
**1TO1**	-12.74	-11.64
**5SV3**	-12.55	-8.00
**3K1R**	-12.28	-11.60
**1UUZ**	-12.27	-11.70
**1Y4A**	-12.17	-11.73
**1Y4D**	-12.16	-11.85
**6NE2**	-12.12	-9.93
**1Y33**	-11.96	-12.72
**3KJ1**	-11.87	-9.80
**5J56**	-11.58	-10.01
**6F2G**	-11.47	-9.66
**3C4P**	-11.40	-10.82
**3BX1**	-11.39	-13.01
**5M2O**	-11.38	-10.59
**3C4O**	-11.37	-11.68
**2Z8W**	-11.36	-10.96

**6FU9**	-11.35	-9.76
**1OPH**	-11.32	-11.56
**1XG2**	-11.32	-10.02
**5K39**	-11.32	-9.46
**2AQ1**	-11.26	-8.39
**1TA3**	-10.98	-10.93
**2I26**	-10.94	-11.60
**1ZV5**	-10.91	-11.50
**1SV0**	-10.85	-5.35
**6E3J**	-10.72	-9.23
**2P42**	-10.59	-11.52
**6JHW**	-10.57	-10.44
**6SAK**	-10.24	-9.40
**4DH2**	-10.21	-9.09
**1P6A**	-10.17	-11.72
**1PK1**	-9.91	-6.89
**6ERE**	-9.73	-7.42
**2UYZ**	-9.67	-7.39
**1MCV**	-9.56	-13.18
**2P45**	-9.46	-11.70
**4PAS**	-9.41	-9.12
**3L33**	-9.37	-10.91
**4U30**	-9.35	-10.50
**3TZ1**	-9.35	-6.81
**4POU**	-9.28	-11.99
**1S0W**	-9.24	-10.12
**6F0F**	-9.20	-8.92
**3BTH**	-9.17	-12.53
**6HUL**	-8.89	-10.41
**4U32**	-8.76	-12.56
**1DZB**	-8.57	-11.00
**3ONA**	-8.41	-9.95
**2IY1**	-8.33	-12.38
**6GHO**	-8.32	-13.08
**5VMO**	-8.26	-9.03
**5NPO**	-8.21	-10.90
**1C1Y**	-8.07	-7.63
**1UUG**	-8.03	-13.52
**3QQ8**	-7.94	-9.10

**2MCN**	-7.80	-8.16
**1LP1**	-7.77	-8.26
**6ISC**	-7.66	-10.11
**4C4K**	-7.60	-8.37
**6FUZ**	-7.49	-9.82
**2IYB**	-7.43	-11.54
**3BTT**	-7.37	-12.20
**4XXW**	-7.22	-11.23
**1L0Y**	-7.12	-7.43
**2C7N**	-7.08	-10.85
**1Z0K**	-6.98	-11.70
**1F3V**	-6.97	-8.95
**2NBV**	-6.86	-7.73
**4H5S**	-6.69	-10.29
**6NRW**	-6.32	-7.19
**4GLA**	-6.07	-10.33
**4A49**	-5.97	-9.11
**1M9E**	-5.70	-6.73
**3F1P**	-5.35	-12.48
**6BA6**	-5.17	-10.59
**1YD8**	-5.10	-9.37
**3N06**	-3.42	-11.95
**3MZG**	-3.14	-12.14
**3NCC**	-0.92	-12.56

Table S3. Comparison of the recent study with representative machine learning-based protein–protein binding affinity prediction models reported in the literature.

Model	Pearson (r)	Reported Error Metric (kcal/mol)	Test Set Size	Evaluation Strategy	Published year

ISLAND[5]	0.44	2.56 RMSE	39	External test	2020
PPI-Affinity[6]	0.62	1.80 MAE	144	Random train-test split	2022
ANN[7]	0.45	1.66 RMSE	39	External test	2023
ProAffinity-GNN[8]	0.67	1.50 MAE	161	External test	2024
**GULP**	**0.54**	**2.31 MAE**	**98**	**Similarity-aware external test**	**2026**

Table S4. Ablation analysis of GNN embeddings and physicochemical features on external test set performance.

Model	Pearson (r)	Spearman (rho)	MAE (kcal/mol)
Physicochemical features only*	0.42	0.46	2.41
GNN embedding only	0.37	0.45	2.62
**GNN embedding + all features**	**0.53**	**0.57**	**2.34**
GNN embedding + top 3 features†	0.42	0.53	2.46
GNN embedding + interfacial features only	0.46	0.55	2.39
GNN embedding + NIS features only	0.46	0.51	2.55

***Linear regression model trained using seven physicochemical descriptors. The fitted model is: y = 7197.6584- 0.0556*IC- 0.0083*HB+ 0.0029*BSA- 0.0598*SB- 72.1055*%NIS_polar- 72.0412*%NIS_charged- 72.0293*%NIS_apolar**

†Top three physicochemical descriptors with the highest absolute Pearson correlation to experimental binding affinity in the training set: IC, HB, and %NIS_polar

Section 3: Model details

Structure preprocessing. Raw PDB files were downloaded from RCSB PDB[9], and were preprocessed using an automated pipeline before feature extraction and model training. For each complex, the biological assembly was first identified from REMARK 350, and only chains assigned to BIOMOLECULE 1 were retained. For NMR structures containing multiple models, only MODEL 1 was kept. All HETATM records, including ligands, ions, and water molecules, were removed, and only standard protein ATOM

records were retained. To avoid ambiguity from alternate conformations, only atoms with alternate location identifier A or blank altloc were kept. The resulting structure was then validated to ensure that exactly two protein chains remained after cleaning. Missing heavy atoms were added using PDBFixer[10], followed by addition of hydrogen atoms at pH 7.0. The final processed structures were written as cleaned, protonated PDB files and used for downstream feature extraction and graph construction.

Graph Construction. Each complex was represented as a residue-level graph using PyTorch Geometric[11], where nodes correspond to Cα atoms and edges were defined based on inter-residue distance thresholds. Residues were assigned to one of three zones based on their structural relationship to the binding interface. Interface residues were defined as those with at least one heavy-atom cross-chain contact within 5.5 Å. Surface-exposed residues with relative solvent accessibility (RSA) ≥ 0.05 were classified as non-interacting surface (NIS) residues. Among NIS residues, those within 15 Å of any interface Cα atom were assigned to the NIS shell zone, while the remaining surface residues were classified as NIS distal. Buried residues (RSA < 0.05) that were not part of the interface were excluded from the graph. Each node was encoded using a 29-dimensional feature vector comprising: a 20-dimensional one-hot amino acid identity, a 3-dimensional residue class encoding (charged, polar, apolar), a 3-dimensional zone one-hot encoding (interface, NIS shell, NIS distal), and a 3-dimensional shell weight vector which reflect the relative contribution of interface, shell, and distal regions. Edges were constructed selectively by zone. Interface–interface edges were added for residue pairs with Cα - Cα distances ≤ 8 Å. NIS shell edges were added for pairs within 12 Å.

Model Hyperparmeter. The graph neural network (GNN) model was constructed using PyTorch[12]. The model consisted of two GCNConv layers (hidden dimension 128), followed by global mean pooling and a two-layer MLP head (128 hidden units with ReLU activation). Node features comprised either 23-dimensional interface features or 29-dimensional NIS-aware features, combined with global physicochemical descriptors. The model was trained using the Adam optimizer (learning rate 1×10^{-3}, weight decay 1×10^{-4}) with batch sizes of 16 (training) and 32 (evaluation). Training employed a weighted mean squared error loss (LOSS_MEAN = −9.9, LOSS_SCALE = 3.0) to emphasize extreme binding affinities, with early stopping (patience = 25, minimum delta = 1×10^{-4}) over a maximum of 300 epochs during cross-validation. Final models were retrained for up to 800 epochs using a ReduceLROnPlateau scheduler (factor = 0.5, patience = 20, minimum learning rate = 1×10^{-5}). Model performance was evaluated using 10-fold cross-validation (random seed = 0).

Training Protocol. The model was trained using the Adam optimizer (learning rate 1×10^{-3}, weight decay 1×10^{-4}) with early stopping (patience = 25 epochs) to prevent overfitting. To address the overrepresentation of moderate-affinity complexes in the training data, we used a weighted mean squared error loss that assigns higher penalty

to predictions on strong or weak binders. To prevent data leakage during cross-validation, we ensured that sequence-similar complexes were never split across training and validation folds. Structures were clustered by pairwise sequence similarity using Smith-Waterman alignment with BLOSUM62 scoring. These clusters were distributed into 10 balanced folds, guaranteeing that no homologous pair was evaluated against its own training context. After cross-validation, the final model was retrained on the full training set and the best checkpoint was saved for inference.

References

(1) Cock, P. J. A.; Antao, T.; Chang, J. T.; Chapman, B. A.; Dalke, A.; Friedberg, I.; Hamelryck, T.; Kauff, F.; Wilczynski, B.; De Hoon, M. J. L. Biopython: Freely Available Python Tools for Computational Molecular Biology and Bioinformatics. *Bioinformatics* **2009**, *25* (11), 1422–1423. https://doi.org/10.1093/bioinformatics/btp163.
(2) Michaud-Agrawal, N.; Denning, E. J.; Woolf, T. B.; Beckstein, O. MDAnalysis: A Toolkit for the Analysis of Molecular Dynamics Simulations. *Journal of Computational Chemistry* **2011**, *32* (10), 2319–2327. https://doi.org/10.1002/jcc.21787.
(3) Gowers, R. J.; Linke, M.; Barnoud, J.; Reddy, T. J. E.; Melo, M. N.; Seyler, S. L.; Domański, J.; Dotson, D. L.; Buchoux, S.; Kenney, I. M.; Beckstein, O. MDAnalysis:

A Python Package for the Rapid Analysis of Molecular Dynamics Simulations. *SciPy 2016* **2016**. https://doi.org/10.25080/Majora-629e541a-00e.
(4) Mitternacht, S. FreeSASA: An Open Source C Library for Solvent Accessible Surface Area Calculations. *F1000Res* **2016**, *5*, 189. https://doi.org/10.12688/f1000research.7931.1.
(5) Abbasi, W. A.; Yaseen, A.; Hassan, F. U.; Andleeb, S.; Minhas, F. U. A. A. ISLAND: In-Silico Proteins Binding Affinity Prediction Using Sequence Information. *BioData Mining* **2020**, *13* (1), 20. https://doi.org/10.1186/s13040-020-00231-w.
(6) Romero-Molina, S.; Ruiz-Blanco, Y. B.; Mieres-Perez, J.; Harms, M.; Münch, J.; Ehrmann, M.; Sanchez-Garcia, E. PPI-Affinity: A Web Tool for the Prediction and Optimization of Protein–Peptide and Protein–Protein Binding Affinity. *J. Proteome Res.* **2022**, *21* (8), 1829–1841. https://doi.org/10.1021/acs.jproteome.2c00020.
(7) Ferraz, M. V. F.; Neto, J. C. S.; Lins, R. D.; Teixeira, E. S. An Artificial Neural Network Model to Predict Structure-Based Protein–Protein Free Energy of Binding from Rosetta-Calculated Properties. *Phys. Chem. Chem. Phys.* **2023**, *25* (10), 7257–7267. https://doi.org/10.1039/D2CP05644E.
(8) Zhou, Z.; Yin, Y.; Han, H.; Jia, Y.; Koh, J. H.; Kong, A. W.-K.; Mu, Y. ProAffinity-GNN: A Novel Approach to Structure-Based Protein–Protein Binding Affinity Prediction via a Curated Data Set and Graph Neural Networks. *J. Chem. Inf. Model.* **2024**, *64* (23), 8796–8808. https://doi.org/10.1021/acs.jcim.4c01850.
(9) Berman, H. M.; Westbrook, J.; Feng, Z.; Gilliland, G.; Bhat, T. N.; Weissig, H.; Shindyalov, I. N.; Bourne, P. E. The Protein Data Bank. *Nucleic Acids Res* **2000**, *28* (1), 235–242. https://doi.org/10.1093/nar/28.1.235.
(10) Eastman, P.; Swails, J.; Chodera, J. D.; McGibbon, R. T.; Zhao, Y.; Beauchamp, K. A.; Wang, L.-P.; Simmonett, A. C.; Harrigan, M. P.; Stern, C. D.; Wiewiora, R. P.; Brooks, B. R.; Pande, V. S. OpenMM 7: Rapid Development of High Performance Algorithms for Molecular Dynamics. *PLOS Computational Biology* **2017**, *13* (7), e1005659. https://doi.org/10.1371/journal.pcbi.1005659.
(11) Fey, M.; Lenssen, J. E. FAST GRAPH REPRESENTATION LEARNING WITH PYTORCH GEOMETRIC. **2019**.
(12) Paszke, A.; Gross, S.; Massa, F.; Lerer, A.; Bradbury, J.; Chanan, G.; Killeen, T.; Lin, Z.; Gimelshein, N. PyTorch: An Imperative Style, High-Performance Deep Learning Library.
